# GPS-telemetry unveils the regular high-elevation crossing of the Himalayas by a migratory raptor: implications for definition of a “Central Asian Flyway”

**DOI:** 10.1038/s41598-020-72970-z

**Published:** 2020-09-29

**Authors:** Nishant Kumar, Urvi Gupta, Yadvendradev V. Jhala, Qamar Qureshi, Andrew G. Gosler, Fabrizio Sergio

**Affiliations:** 1 Edward Grey Institute of Field Ornithology, University of Oxford, Zoology Research and Administration Building, 11a Mansfield Road, Oxford, OX1 3SZ UK; 2 Mansfield College, University of Oxford, Mansfield Road, Oxford, OX1 3TF UK; 3 Wildlife Institute of India, Post Box # 18, Chandrabani, Dehradun, Uttarakhand 248001 India; 4 Institute of Human Sciences, School of Anthropology and Museum Ethnography, 58a Banbury Rd., Oxford, OX2 6QS UK; 5 Department of Conservation Biology, Estacion Biologica de Doñana - CSIC, C/Americo Vespucio, 26, 41092 Sevilla, Spain

**Keywords:** Ecology, Animal behaviour

## Abstract

Remote technologies are producing leapfrog advances in identifying the routes and connectivity of migratory species, which are still unknown for hundreds of taxa, especially Asian ones. Here, we used GPS-telemetry to uncover the migration routes and breeding areas of the massive population of migratory Black-eared kites wintering around the megacity of Delhi-India, which hosts the largest raptor concentration of the world. Kites migrated for 3300–4800 km along a narrow corridor, crossing the Himalayas at extremely high elevations (up to > 6500 m a.s.l.) by the K2 of the Karakoram Range and travelled long periods at elevations above 3500 m. They then crossed/circumvented the Taklamakan Desert and Tian Shan Range to reach their unknown breeding quarters at the intersection between Kazakhstan, Russia, China and Mongolia. Route configuration seemed to be shaped by dominant wind support and barrier avoidance. Wintering ranges were smaller than breeding ranges and concentrated around Delhi, likely in response to massive human food-subsidies. Our results illustrate that high-elevation crossings by soaring migrants may be more common than previously appreciated and suggest the delineation of a hitherto poorly-appreciated “Central Asian Flyway”, which must funnel hundreds of thousands of migrants from central Asia into the Indian subcontinent via multiple modes of the Himalayan crossing.

## Introduction

Identifying migration routes and the connectivity between breeding and non-breeding areas of migratory species is crucial for their conservation, as threats operating during migratory journeys, at stopover sites and in the non-breeding quarters may have marked repercussions on the survival rates of individuals and the persistence of their populations^1–3^. Remote technologies, such as biologging, are currently providing unprecedented insights into migratory strategies and their population consequences. However, main migration routes are still unknown for most species and over broad geographic areas. In particular, while many remote-tracking studies have focused on species breeding in North America and the western Palearctic, extremely few detailed data exist for species using Asian flyways (e.g.^4–7^) and most information is still based on route reconstructions hypothesized on the basis of scanty recoveries of ringed birds, or observations at sites that concentrate passing migrants (e.g.^8–10^). Information on Asian flyways is additionally important because hundreds of species which breed in central-northern Asia spend their non-breeding season in the Indian sub-continent and tropical countries of south-eastern Asia, all of which are experiencing rapid anthropogenic habitat loss and urbanization^11,12^. This will likely affect the population health of migrants and their potential to spread infectious diseases dangerous for humans, such as avian influenza^13,14^, particularly in the case of species that are urban human commensals and thus in constant close contact with dense human populations. Thus, delineating the migration routes of such human commensals entails additional urgency.

Finally, one of the least known aspects of the Asian migration system is how soaring migrants travelling to and from the Indian subcontinent cope with the major obstacle represented by the Himalayan range. While recent studies have extensively uncovered the barrier-passage routes of birds that migrate mainly by flapping flight, such as ducks and geese (e.g.^5,7,15,16^), detailed GPS-tracking data for soaring migrants are still almost non-existent, except for a study on a single Steppe eagle *Aquila nipalensis* individual^6^.

Here, we focus on a raptorial human commensal, the Black kite *Milvus migrans*, and illustrate for the first time the migration routes, breeding and non-breeding ranges of the population that winters within and around the city of Delhi (India), thus contributing to generate the largest raptor concentration of the world^17–19^. The Indian subcontinent hosts two main Black kite subspecies: *M. m. govinda* or Small Indian kite, which is a resident or semi-resident breeder nesting almost exclusively in urban settings, and *M. m. lineatus *or Black-eared kite, which is a migratory visitor from its northern breeding areas in central-northern Asia^20,21^. The exact provenance and migration routes of the *lineatus* individuals that spend their non-breeding months in India are virtually unknown, except for a single ring-recovery at the end of October 2001 in North-eastern India (at the border with Myanmar; 24.36°N, 93.54°E), just south of the easternmost Himalayas, of a bird ringed in south-central Mongolia (44.25°N, 105.19°E)^22^. The current knowledge of the migratory and wintering patterns of *lineatus* kites in India is further muddled by several reports of large winter roosts of Black-eared kites near rubbish dumps or in urban settings misidentified as Small Indian kites, given the complexity of separating the two subspecies, especially when looking at hundreds and sometimes thousands of individuals at once (e.g.^23,24^).

## Results

**Figure 1:**
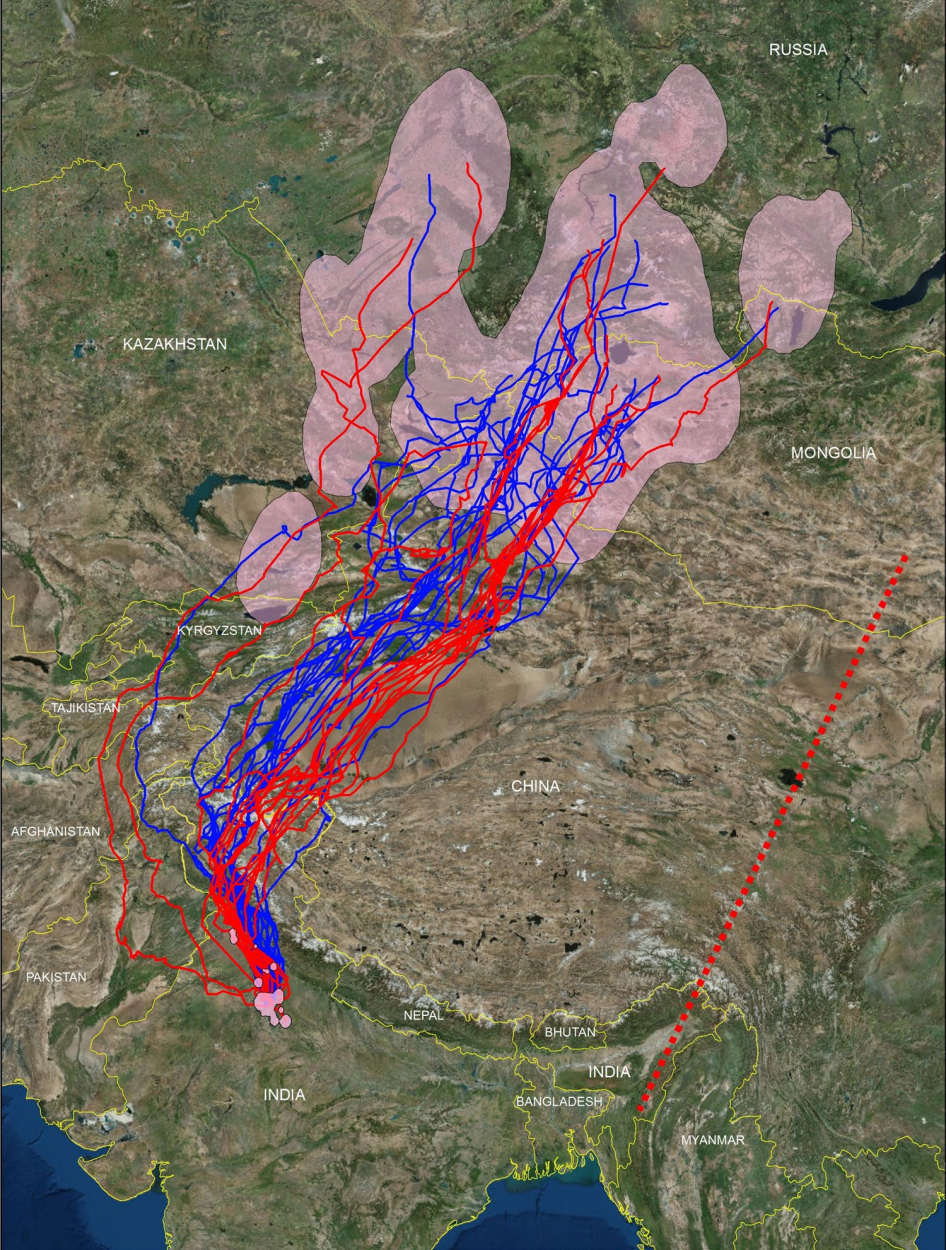
Pre-breeding (blue tracks) and post-breeding (red tracks) migration routes, and breeding and non-breeding ranges (pink polygons) of Black-eared kites *Milvus migrans lineatus* GPS-tagged in Delhi (India). The polygons of the ranges represent 99% Kernels built on the cumulative sum of the GPS-locations of all tagged individuals. The broken red line connects the likely breeding and wintering locations of a *lineatus* kite, based on a ring recovery in North-East India, bordering Myanmar^21^. This map was generated with ArcGIS 10.5, using World Imagery by Esri, Maxar, GeoEye, Earthstar Geographics, CNES/Airbus DS, USDA, USGS, AeroGRID, IGN and the GIS User Community (https://services.arcgisonline.com/ArcGIS/rest/services/World_Imagery/MapServer), and country borders according to Google Earth imagery (Google India).

The migration tracks of all tagged kites are shown in Fig. 1. There was no evidence of kites migrating by night. Pre-breeding and post-breeding migrations largely overlapped: kites crossed the Himalayas in its western portion (Trans-Himalayas), traversed or circumvented the Taklamakan Desert of the Xinjiang Province and the Tian Shan Mountain Range of China, ending up in a wide region centred on the Altai Mountain Range at the intersection of western Kazakhstan, southern-central Russia, western Mongolia and North-western China (Fig. 1). In three cases (one northbound and two southbound migrations), kites took an even more westerly route which brought them to the same destination through eastern Pakistan, Afghanistan, Tajikistan and Kyrgyzstan (Fig. 1). Overall, the tagged kites travelled relatively fast, completing a 3300–4800 km journey in 13–47 days, progressing on average by 150–240 km/day, with significantly faster speeds in the pre-breeding migration (Table 1).

**Table 1:** Estimates of pre-breeding and post-breeding migration by Black-eared kites GPS-tagged in Delhi during 2014–2018.

Migration component	N^a^	Pre-breeding migration Mean ± SE (range)	N^a^	Post-breeding migration Mean ± SE (range)	*t*	P
Departure date^b^	20 (16)	104.15 ± 11.57 (75—126)	17 (14)	243.65 ± 21.78 (224—258)		
Arrival date^b^	20 (16)	126.25 ± 8.12 (97—155)	17 (14)	270.29 ± 40.43 (255—290)		
Speed (km/day)	17 (14)	198.98 ± 14.82 (77.02—267.89)	14 (12)	151.00 ± 12.56 (71.36—253.91)	2.41	**0.023**
Active speed (km/travelling day)	17 (14)	236.59 ± 7.99 (182.99—302.42)	14 (12)	198.52 ± 8.34 (155.14—276.99)	3.28	**0.003**
Duration (days)	17 (14)	22.82 ± 2.67 (13—45)	14 (12)	27.14 ± 2.88 (12—47)	1.08	0.281
Travelling days	17 (14)	17.00 ± 0.94 (13—25)	14 (12)	19.00 ± 1.33 (11—27)	1.26	0.218
Number of stopovers Days of stopover	17 (14) 17 (14)	1.65 ± 0.34 (0—5) 5.82 ± 2.03 (0—28)	14 (12) 14 (12)	2.64 ± 0.41 (0—5) 8.14 ± 1.98 (0—28)	1.88 0.81	0.071 0.426
Route length (km)	17 (14)	3926.56 ± 108.37 (3333.1- 4620.5)	14 (12)	3668.18 ± 151.68 (2963.71—4767.74)	1.42	0.167

^a^Number of migration episodes (number of tagged individuals).

^b^Julian date (1 = 1 January).

Differences in mean values were tested by means of a t-test and significant results are highlighted in bold.

The mean area of individual home ranges was larger during the breeding season (57,515 ± 30,151 km^2^) than in the non-breeding Indian range (195 ± 59 km^2^; Mann–Whitney U test = 55, P < 0.05). Similarly, the cumulative range of all tagged individuals was 85-fold larger in the breeding quarters (1,520,099 km^2^) than in the Indian wintering quarters (17,898 km^2^). In India, all individuals were constantly located in highly anthropogenic habitats, almost invariably in the vicinity of rubbish dumps, abattoirs, slaughterhouses and other animal processing facilities. On the contrary, breeding ranges were mainly located in natural or semi-natural habitats: out of 20 identified breeding ranges by 16 individuals, 13 were dominated by extensive farmland, 5 by arid—steppe habitats and one by marshland (based on inspection of Google Earth imagery). Interestingly, one of the four kites that could be tracked for two consecutive breeding periods (data are still accumulating for the rest) radically changed its range from one year to the next, moving from North-western China to a site some 1100 km away in South-central Russia, which may suggest dependence on ephemeral resources.

## Discussion

The GPS-tagged kites accomplished a 3300–4700 km migration that connected their non-breeding quarters around the trapping site in Delhi to a rather compact and continuous breeding range at the intersection between western Kazakhstan, Northern China, western Mongolia and southern Russia. The route was similar between the pre- and post-breeding migrations and ran as a relatively narrow corridor that passed through several important successive macro-barriers, such as the Himalayas, the Taklamakan Desert of southern China and the Tian Shan mountains.

In both northbound and southbound migrations, kites did not cross the Himalayas through the most direct route to-from their breeding grounds, but rather made a detour of 400–1000 km north-west of Delhi to cross the Himalaya barrier between Punjab and Kashmir. A few individuals made an even more western detour through Pakistan and Afghanistan and thus almost completely avoided the extremely high crossings of the Himalayas. However, most kites crossed the Himalayas by the Karakoram mountain range, several of them by its highest K2 peak at extremely high altitudes of up to more than 6500 m a.s.l., as recorded by the on-board GPS and as confirmed by the terrain below. Furthermore, many individuals covered stretches of more than 500 km with unavoidably continuous elevations of > 3500 m a.s.l., thus spending long periods at high altitudes. Overall, such crossings must require special physiological adaptations^25^ and had been mainly documented as a common population strategy for larger species (e.g. large eagles) or for reputedly more powerful fliers and non-soaring migrants such as falcons, ducks and geese (e.g.^5,15,16^). The western crossing of the Himalayas was thus unlikely to be caused by mere high-elevation avoidance and was probably determined by some interaction between wind-support and minimization of barrier-threats. In Appendix 1 we show the average wind directions for the main months of migration. Two aspects of dominant winds seem important: (1) direct crossing of the Himalayas by the shortest route from Delhi would expose kites to strong south-westerly winds once on the Tibetan Plateau, which would displace them eastwards, thus causing a subsequent unavoidable 1500–2000 km of continuous journey over high-elevation or desert lands to reach the breeding grounds; (2) on the contrary, dominant winds are less strong and more favourably oriented in the western Himalayas—western Taklamakan Desert, and such route allows (2a) the western circumvention of the desert through large patches of irrigated farmland and (2b) the eastern circumvention of the Tian Shan mountains (with peaks > 7000 m high and elevations often > 3500 m). A more quantitative analysis of wind-and barrier-effects will be presented elsewhere.

Negotiation of wind support and geophysical barriers led to migratory speeds comparable or even higher than those reported in other telemetry studies^2^, and for the nominal subspecies migrating along the west European-west African flyway with markedly less dangerous obstacles^26,27^. Thus, whatever strategies were in place seemed to afford clear advantages as they led to journeys as efficient as those reported for easier routes. Curiously, in the latter Euro-African study, the post-breeding migration was shorter and more rapid than the pre-breeding migration^26^, while the opposite was evident in the current Asian study. Such difference is congruent with variation in wind conditions between the two flyways: in the west European—west African flyway, strong headwinds and tailwinds respectively characterize the pre-breeding and post-breeding migration, while wind conditions in the Asian flyway are rather similar between autumn and spring. By exclusion, the higher Asian speeds for the northward migration were likely propelled by the urgency to arrive early at the breeding quarters and the fitness benefits that this ensures in kites and other species (e.g.^26–29^, review in^2,30^). This comparison underlines how migration dynamics may change radically between different flyway-systems in response to the delicate balance between barrier negotiation and weather support, even within a single species. This suggests caution in generalizations from studies conducted at a single flyway and underscores the need for more research from the very poorly known Asian flyways.

**Figure 2:**
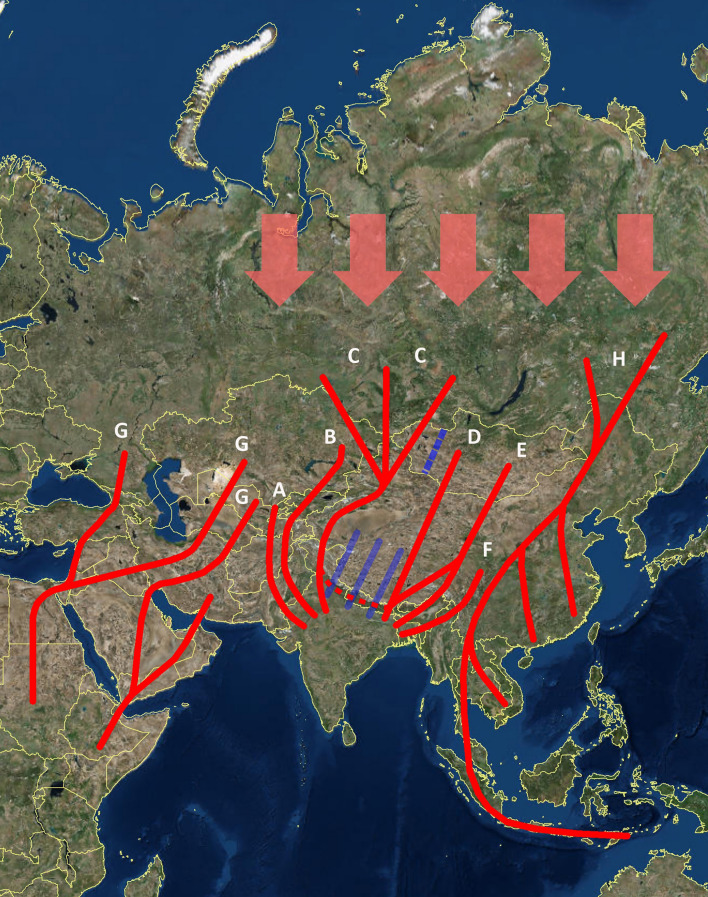
Main Asian flyways (red lines) used by soaring migrants in Asia during pre- and post-breeding journeys. Traditional flyways-charts (e.g.^2,31^) depicted two major flyways for Asia: the Eurasian—East African flyway (G) and the East Asian Continental flyway (H). We propose the addition of a composite “Central Asian flyway” (routes A to F) employed by soaring migrants from central Asia to enter or exit their non-breeding quarters in the Indian sub-continent. The passage of soaring migrants is likely to be mainly funnelled through the westernmost (**A**, **B** and **C**) and easternmost (**D**, **E** and **F**) ends of the Himalaya Range. The eastern crossing of the Himalayas has been poorly documented by tracking-studies for soaring migrants and thus three hypothetical nonexclusive routes are shown. The connection between route D and E is supported by satellite-tracking data on a single Steppe Eagle by Batbayar and Lee^6^. Plotted routes B and C are based on data from this study, while route A is based on tracking data by Juhant & Bildstein^10^ and Terraube and colleagues^40^. Based on terminology proposed in Juhant & Bildstein^10^ and new routes outlined in this study, we define: route A and B as the “Western Circum-Himalayan Corridor”; route C as the “Western Himalayan Corridor”; route D and E as the “Eastern Himalayan Corridor”; and route F as the “Eastern Circum-Himalayan Corridor”. The red dashed line represents a West–East migration corridor reported by several authors for several raptors, especially for the autumn migration (western Himalaya^9^; eastern Himalaya^6,41,42^). It may represent the strategic, approaching movement to the Himalaya crossing in the pre-breeding migration, or the post-crossing exploitation of favourable winds in the post-breeding migration. More tracking data from more species will be needed to uncover its function. The blue dashed line in western Mongolia indicates a likely migratory divide: species passing to the west/east of such divide seem to use the western/eastern Himalayan routes, respectively. This divide also seems to hold for other non-soaring taxa, such as ducks and geese (e.g.^5,15,16^). The passage of species capable of migrating trough more direct flapping flight or through mixed strategies based on both soaring and flapping (e.g. falcons, harriers, Accipiter hawks) may employ a more central, broad-range crossing of the Himalayas (conceptually approximated by continuous blue lines over a portion only of the Himalaya range to avoid map clogging, (e.g.^7,15^). The thick red arrows at the top of the map (central-northern Russia) represent the theoretical, more northern basin of provenance of migrants travelling through the more southern depicted routes in the outward, post-breeding migration. The map integrates data from the current study, tracking data from other studies (e.g.^5–7,16,40^), re-sightings of ringed birds (e.g.^22^), observations at watch-sites of concentrated passage by migrants (e.g.^8–10,41,42^) and previous large-scale maps of main flyways for soaring birds^2,31^. Major routes are shown only up to their main entry-areas into the Indian subcontinent and into Africa. This map was generated with ArcGIS 10.5, using World Imagery by Esri, Maxar, GeoEye, Earthstar Geographics, CNES/Airbus DS, USDA, USGS, AeroGRID, IGN and the GIS User Community (https://services.arcgisonline.com/ArcGIS/rest/services/World_Imagery/MapServer), and country borders according to Google Earth imagery (Google India).

For example, up to date two major flyways have been reported for Asian raptors and other soaring migrants^2,31^ (Fig. 2): a western route leading from central-western Russia and Kazakhstan to eastern Africa (usually called Eurasian-East African flyway) and an eastern route leading from eastern Russia—Japan to south-eastern Asia, Indonesia and Australia (usually called East Asian continental flyway). This leaves out what can be defined as a “Central Asian flyway”, well appreciated for non-soaring migrants such as ducks and geese (e.g.^5,17^) but not for the massive number of soaring birds that mast cross the Himalayas each year. This flyway is composed of a series of corridors (routes A-F in Fig. 2), that must funnel hundreds of thousands of soaring migrants from Kazakhstan, central-northern Russia, Mongolia and central-northern China into the Indian subcontinent and vice-versa. Soaring migrants are likely to concentrate their crossings at the westernmost and easternmost ends of the Himalayan range and Tibetan plateau, as shown by our data, other satellite data from a single eagle^6^, and casual observations (e.g.^22^), while “flapping” migrants may also cross through the central sectors of Nepal^5,7^ (e.g. blue lines in Fig. 2; see also detailed multi-species maps in^15^). These entries or exit routes could be defined as the “Western Circum-Himalayan Corridor” (route A and B in Fig. 2); the “Western Himalayan Corridor” (route C); the “Eastern Himalayan Corridor” (route D and E); and the “Eastern Circum-Himalayan Corridor” (route F). Note that the systematic use of the western portion of this central flyway by all our tagged birds would already imply the passage of thousands of Black-eared kites known to winter around the Delhi region alone, in what is probably the greatest raptor concentration of the world. To our knowledge, such massive and funnelled passage at high-elevation was unknown, may be easily representative of the migration dynamics of other soaring species and it may easily have a similar counterpart somewhere in the eastern Himalayas. Finally, the fact that all our tagged kites bred eastward up to western Mongolia and that the few available data for birds migrating from central Mongolia showed a heading towards the easternmost end of the Himalaya range^16,22^ suggest the possibility of a migratory divide in central-western Mongolia for kites and possibly for other species (blue dashed line in Fig. 2). Birds breeding west or east of such divide would be more likely to use the western or eastern Himalaya-crossing of the Central Asian flyway. Similar threshold-divides have been previously reported for other species (e.g.^32^).

Migration connected breeding and non-breeding ranges of very different extent. Both at the individual and cumulative population-level, breeding ranges were way larger than the non-breeding, Indian ranges (Fig. 1). This difference was likely caused by the enormous availability of predictable human food subsidies in the Indian landscape. In the Indian quarters, all tagged kites behaved as specialists of anthropogenic resources: they used landfills and abattoirs almost exclusively, and frequented urban or suburban sites of Delhi and surrounding towns and cities (up to 300 km from Delhi), where they often congregated into flocks of thousands of individuals (up to 10,000 counted by us at a regularly monitored single landfill). This was likely promoted by: (1) the enormous availability of human meat waste in an area of extremely high human density (16 million inhabitants in Delhi alone), ranking as the most important meat-producing area of India^33^ and coupled with poor sanitation infrastructures to process such refuse; (2) the additional availability of meat waste intentionally offered to kites by workers of abattoirs and animal processing facilities as part of the religious practice of offering a service to all divine creations. These cultural beliefs are integral to the extremely benevolent attitudes of most Indians to wildlife, which probably further favoured an urban lifestyle by kites, as previously demonstrated for the locally-resident Small Indian kite subspecies^18,19,34^. Thus, the local availability of enormous human food subsidies likely caused the striking concentrations of thousands of individuals, and the small home ranges and restricted distribution of the tagged kites, which spent all winter in Delhi and surrounding towns. This is in stark contrast with the wintering behaviour of the nominate subspecies in the western Palearctic, whose individuals may range nomadically over several African nations in a single winter^35,36^. It also contrasts with the behaviour of the tagged kites at the breeding quarters, where they ranged over wide areas, avoiding large cities and usually settling in natural or semi-natural habitats with scattered human populations, such as extensive farmland, steppe, arid rangelands, or wetlands. Only five individuals settled in proximity of medium-small towns, but barely ever used them for foraging.

In conclusion, GSM and satellite GPS-telemetry allowed the efficient monitoring of animal movements across nine Asian nations and from sea level up to above 6500 m high elevations, resolving the mystery of the provenance of the massive concentrations of kites observed in the Delhi-region over winter. In this sense, remote bio-logging continues to unveil biological phenomena that were unthinkable of, up until recently. Our results further implied the systematic migratory crossing of the Himalayas at extremely high elevations by thousands of individuals of a species up to now unknown to accomplish such task and not congruent with the type of flight-mode and capabilities previously associated with such undertakings. This markedly broadens the range of species that may be expected to operate similar migratory tactics, an aspect that has received minor attention and clearly deserves more investigation. Finally, the current data imply the existence of hitherto overlooked migration routes likely used by hundreds of thousands of soaring migrants, thus suggesting the necessity of a better appreciation of the “Central Asian flyway” among the current set of major flyways for avian migrants.

## Methods

### Ethics statement

This research was part of a larger and long-term study on the demography and movement ecology of Black kites in Delhi. We received the permits to conduct the fieldwork from the office of the Additional Principal Chief Conservator of Forests (APCCF), the Government of the National Capital Territory of Delhi under the provisions of the Wildlife Protection Act, 1972 (permit number: CF/LC/105/07/HQ/10504-8). The Training, Research, and Academic Council (TRAC) of the Wildlife Institute of India, Dehradun (WII), gave bioethical approval for the research protocols. We performed all methods in accordance with the relevant guidelines and regulations laid out by TRAC WII with respect to our study animals.

### Field procedures and GPS-tagging

We GPS-tagged 19 Black-eared kites (14 adults and five pre-adults) between 2014 and 2018. Kites were trapped at the landfill of Ghazipur in Delhi by means of mist-nests and Bal-chatri traps positioned over carrion baits. Kites were tagged through 25 g GSM-GPRS PTT manufactured by e-obs GMBH (n = 13 individuals) and PTT-100 Solar Argos/GSM/GPS PTTs of 22 g and 25 g manufactured by Microwave Telemetry, Inc. (n = 6). GPS locations had an estimated error of 18 m. Transmitters weighed less than 3% of the body mass and were fitted as backpacks through a Teflon harness with the same method recently used for the nominate subspecies in another study which showed no adverse effects on body condition, foraging performance, breeding output, survival, recruitment and longevity, or on the capability to raise healthy offspring^37^. Furthermore, the larger size and mass of the *lineatus* subspecies implied that a lighter transmitter-load was imposed in the current study.

### Data processing

Given that the duty cycles of the three tag-types varied from 1 GPS-location/hr to over 1000 GPS-locations/day, we down-filtered the data to a uniform sampling interval of one location/hr, so as to obtain estimates comparable to most previous studies and avoid biases caused by pooling data based on differential location sampling regimes^38^. Migration tracks from tags that showed signs of malfunctioning (e.g. with no locations for consecutive days during migration or for consecutive weeks during the breeding or non-breeding period) were discarded from the analyses. However, if these tags recorded enough locations during the period of migratory departure and arrival, data on migration timing was retained for analysis. For this reason, estimates on departure and arrival dates are based on slightly larger sample sizes.

Migrations were characterized by the parameters commonly used in this type of studies: (1) departure date; (2) arrival date; (3) kilometres travelled per day; (4) kilometres travelled per day of active migration (i.e. a day with more than 50 km of directed flight); (5) number of days to complete the journey (stopovers included); (6) number of travelling days; (7) number of stopovers at staging areas (i.e. a day with less than 50 km of directed flight); (8) number of days of stopover at staging areas; and (9) route length. The spatial extent of the home range of each kite during the breeding and non-breeding period was assessed by means of a 99% Kernel Density estimator^39^. Two further Kernel polygons were built after pooling the GPS-locations of all individuals, in order to define the cumulative distribution range of the whole population during the breeding and non-breeding period, respectively. To ensure the comparability of such estimates, home ranges were calculated for the two months in which all individuals were simultaneously present, i.e. June + July for the breeding period and November + December for the non-breeding period. Differences between means were tested by means of t-tests for normal data, or Mann–Whitney U tests for data that did not conform to normality. Statistical significance was set at α < 0.05, and all means are given ± 1 SE. The figures were generated with ArcGIS 10.5, using World Imagery by Esri, Maxar, GeoEye, Earthstar Geographics, CNES/Airbus DS, USDA, USGS, AeroGRID, IGN and the GIS User Community (https://services.arcgisonline.com/ArcGIS/rest/services/World_Imagery/MapServer), and country borders according to Google Earth imagery (Google India).

## Data Availability

Data will be available on reasonable request from the authors.
